# Carbolic Acid as an Antiseptic

**Published:** 1869-11

**Authors:** J. Fred. Jennings

**Affiliations:** Apothecary


CARBOLIC ACID AS AN ANTISEPTIC.—*To the Druggists' Circular:* Thinking that some of the readers of the *Circular* may be benefited by a description of an experiment that I tried on the body of a lady seventy years old, who died July 18th, induces me to submit the following to your notice, and, if thought worthy, to a place in your columns.

The corpse was simply washed with a solution of carbolic acid—strength, one part carbolic acid, cryst., to eighty parts water. No injection was made, nor cut, nor incision on any part of the body. After the body had been washed with the solution, the skin became soft and pliant, the joints perfectly free, the expression good and natural, and the lips resumed a florid and life-like appearance. Parts of the body that had turned dark before the solution had been applied, gradually recovered their color. The corpse lay in a room facing the sun, and although the thermometer stood at 85° F., not the least smell or odor was perceptible. That carbolic acid is the best article for preserving bodies for a few days previous to burial there can be no doubt, and is of too much importance to be cast aside. Hoping you will give the above a place in your columns, I remain,

Your ob't servant,

J. FRED. JENNINGS, Apothecary.

